# Architectural Response of Wheat Cultivars to Row Spacing Reveals Altered Perception of Plant Density

**DOI:** 10.3389/fpls.2019.00999

**Published:** 2019-08-07

**Authors:** Mariem Abichou, Benoit de Solan, Bruno Andrieu

**Affiliations:** ^1^UMR ECOSYS, INRA, AgroParisTech, Université Paris-Saclay, Thiverval-Grignon, France; ^2^ARVALIS – Institut du Végétal, Paris, France; ^3^UMR EMMAH, INRA, Avignon, France

**Keywords:** row spacing effect, wheat, architectural response, tillering, stem angle, leaf angle, space prospection, plasticity

## Abstract

Achieving novel improvements in crop management may require changing interrow distance in cultivated fields. Such changes would benefit from a better understanding of plant responses to the spatial heterogeneity in their environment. Our work investigates the architectural plasticity of wheat plants in response to increasing row spacing and evaluates the hypothesis of a foraging behavior in response to neighboring plants. A field experiment was conducted with five commercial winter wheat cultivars possessing unique architectures, grown under narrow (NI, 17.5 cm) or wide interrows (WI, 35 cm) at the same population density (170 seeds/m^2^). We characterized the development (leaf emergence, tillering), the morphology (dimension of organs, leaf area index), and the geometry (ground cover, leaf angle, organ spreading, and orientation). All cultivars showed a lower number of emerged tillers in WI compared to NI, which was later partly compensated by lower tiller mortality. Besides, the upper leaf blades were larger in WI. Finally the leaf area index at flowering showed little difference between WI and NI treatments. The rate of leaf emergence and the final leaf number were higher in WI compared to NI, except for one cultivar. Around the start of stem elongation, pseudo-stems were more erect in WI, while around the time of flowering, stems were more inclined and leaves were more planophile. Cultivars differed in their degrees of responses, with one appearing to prospect more specifically within the interrow space in WI treatment. Altogether, our results suggest that altering interrow distance leads to changes in the perceived extent of competition by plants, with responses first mimicking the effect of a higher plant density and later the effect of a lower plant density. Only one cultivar showed responses that suggested a perception of the heterogeneity of the environment. These findings improve our understanding of plant responses to spatial heterogeneity and provide novel information to simulate light capture in plant 3D models, depending on cultivar behavior.

## Introduction

Interrow distance is an important aspect of crop management. Generally, the choice of row spacing reflects a compromise between optimizing resource capture by plants and practical constraints, such as allowing space between rows for the mechanical control of weeds in organic farming, or use of seeders adapted to no-till practices. In conventionally grown wheat crops, row spacing varies from 12 to 18 cm, whereas it can vary from 35 to 50 cm in organic or no-till farming conditions. These choices are largely empirical, and understanding how row spacing modulates plant growth and the possible genotypic variability in the responses would facilitate the design of new cultural practices. Changing row spacing obviously impacts interactions between neighboring plants; such interactions involve competition for resources (Kirby and Faris, 1972; Alzueta et al., 2012), as well as active morphogenetic responses triggered by the perception of neighbors. It is widely recognized that red:far-red ratio (R:FR) acts as an early signal promoting plant responses, such as reduction of tillering and faster stem extension (Ballaré and Casal, 2000; Evers et al., 2006). Touch-signals between neighboring plants can also trigger early responses (Ballaré, 1999; de Wit et al., 2012). The increase of row spacing at constant density results in a non-isotropic change in density with an increase in the distance between plants of different rows and a decrease within a row, possibly altering the relationship between perception of neighbors and competition for resources. Interestingly, some species are able to directionally respond to an anisotropic environment. For instance, horizontal gradients of blue light guide dicot plant shoots toward canopy gaps in patchy vegetation (Ballaré, 1999), whereas R:FR signals allow maize plants to avoid their closest neighbors by orientating their leaves perpendicularly to the row direction (Drouet and Moulia, 1997; Maddonni et al., 2002). The existence of similar behavioral responses has not been documented for wheat.

A large amount of work has been dedicated to analyzing the response of wheat yields to sowing density, altered by changing interrow distances and/or interplant distance within a row. They were motivated by a range of questions, including optimizing light capture and competing with weeds, facilitating crop management, and/or dealing with scarcity of water and nutriments. Provided that weeds are efficiently controlled, plasticity allows wheat plants to compensate for large differences in planting density. It has been reported that there is little impact on yield for plant densities ranging from 70 to 400 plants/m^2^, or between 16 and 400 plants/m^2^ when square planting arrangements are used (Darwinkel, 1978; Whaley et al., 2000; Fischer et al., 2019). The usual sowing density (170–250 plants/m^2^) is higher than the value of 70 plants/m^2^ mentioned above, which can be thought of as insurance against the risk of winter mortality and as a means to control weed development (Lemerle et al., 2001, 2004; Weiner et al., 2001; Van Der Meulen and Chauhan, 2017). Responses to density are subject to large Genotype^∗^Environment (G^∗^E) interactions and the classical work of Donald (1968) defined ideotypes adapted to high density (short stems, few tillers, and erect leaves) or to low density (high tillering and prostrate leaf stature). Donald’s ideotypes, however, appeared to account only partly for cultivar responses to density: indeed it was recognized that the higher yielding cultivars at high density lose their advantage at low density (Reynolds et al., 1994), but a superiority at low density of highly tillering cultivars with a prostrate leaf stature was not clearly confirmed experimentally (Araus et al., 1993; Reynolds et al., 1994), except may be for very low density (Fischer et al., 2019). Sadras and Lawson (2011) proposed that performance at low density is more linked to the plasticity of traits than to the value of traits shown at normal density. All of the aforementioned studies report tillering as the major determinant of plasticity, besides – to a lower degree – there is also an increase in the size of upper leaves at low density (Dornbusch et al., 2011).

Changing density by increasing interrow distance was reported by several authors (e.g., De Vita et al., 2017; Fischer et al., 2019) to have a stronger impact than changing the density of plants within a row, as it leads to increased crop spatial heterogeneity. Several studies have specifically investigated the effects of varying row spacing, while maintaining a same plant density, on yield. The potential benefit of reducing the interrow distance below the conventional values has been investigated mostly with the objective of increasing production in conventional agriculture. Most authors reported that narrow interrows allowed for an increase in yield (Mülle and Heege, 1981; Frederick and Marshall, 1985; Joseph et al., 1985; Marshall and Ohm, 1987; Johnson et al., 1988; Epplin et al., 1992; Deswarte and Gouache, 2011; Liu et al., 2016). Several reports identified associations linking increases in yield with increases in ear number. The amount of increase is subject to strong G^∗^E interactions (Marshall and Ohm, 1987). In contrast, there are also a significant number of studies that report a lack of effect (Freeze and Bacon, 1990; Teich et al., 1993; Lafond, 1994; Lafond and Gan, 1999; Chen et al., 2010). The impact of wide row spacing has primarily been investigated in the context of no-till or organic farming, in which yield potential is usually below that of conventional practices. In these conditions, most authors report that wide row spacing results in yields similar to those from conventional spacing (Lafond, 1994; Lafond and Gan, 1999) or slightly higher (McLeod et al., 1996; Hiltbrunner et al., 2005). Wide row spacing generally results in a lower density of ears, but this is compensated by a higher number of kernels per ear and higher individual kernel weights. Hiltbrunner et al. (2005) also reported higher concentrations of grain protein under wide row spacing. In high yielding conditions (7–12 tons/hectare), Deswarte and Gouache (2011) reported significant decreases in yield with larger row spacing, with marked differences in the level of decrease depending on the cultivar.

Finally, it appears that wheat plasticity largely compensates for differences in interrow distance within the range of 7–40 cm, and only in high yielding conditions is there a recognized trend of decreasing yield with increasing interrow distance. The previous studies mentioned above suggest significant Genotype^∗^Interrow interactions. It would seem logical to expect that genotypes with upright leaves are the most capable of benefiting from narrow row spacing, while a spreading growth phenotype would enhance resource capture under wide row spacing. Deswarte and Gouache (2011) mentioned some observations in support of this idea, but without quantitative assessment. On the other hand, when comparing a range of cultivars, Marshall and Ohm (1987) did not find associations between simple traits and the ability to maintain yield under different interrow spacing. Sadras and Lawson (2011) suggested that characterizing plasticity is more important that the standard value of traits for understanding the response to density. Except for the consistent effect on tillering and ear number, there is little information on the plasticity of shoot architecture in response to interrow distance.

The aim of the present work was to document how wheat plants adapt their development, architecture, and geometry in response to increasing row spacing while maintaining constant population density. We focused on aerial architecture and addressed three principal questions: (i) Could we identify consistent views on how interrow distance impacts tillering dynamics, organ dimensions, and other aspects of shoot development? (ii) Are wheat plants able to modify their shoots to explore the free space between rows? (iii) Are there differences in responses between cultivars? Addressing these questions may help to identify or design cultivars adapted to new crop management regimes and more generally to improve our understanding of the adaptive changes in plant architecture that may exist when growth occurs in anisotropic environments.

## Materials and Methods

The development, architecture, geometry, and yield of five winter wheat cultivars were characterized in field conditions using two contrasting row spacing systems. The five cultivars were chosen to broadly represent a range of characteristics including the ability to cover the soil and the nature of leaf stature (from erectophile to planophile). Data were analyzed to assess differences existing at plant and crop scale between row spacing treatments, and identify possible differences in behavior between cultivars. The data leading to the results presented are provided as Supplementary Data Sheet S2.

### Experimental Design and Treatments

Experiment were conducted in 2012 and 2013 at the INRA campus of Thiverval-Grignon (48°51 N, 1°58 E), with a maritime influenced climate and a deep loamy soil. Five cultivars of winter wheat *Triticum aestivum* (Caphorn, Apache, Maxwell, Soissons, and Renan) were sown on October 2, 2012. The cultivar Caphorn (issued in 2000) is adapted to conventional practices and characterized by short stems with upright upper leaves, whereas Apache (1998) and Renan (1989) are well adapted to organic farming and have a higher ability to cover ground due to their tillering capacity (Apache) or planophile stature (Renan). Maxwell (2007) and Soissons (1988) represent intermediate types. Field treatments consisted of two blocks (15 m × 1.75 m) per treatment. The distance between rows was either 17.5 cm, which is the conventional distance in the region (narrow row spacing, NI), or 35 cm (wide row spacing, WI). The mean inter-seed distance along the row was 2.9 cm in NI treatments and 1.4 cm in WI treatments, ensuring a same density of 170 seeds/m^2^ in all cases. After emergence, we suppressed some seedlings to compensate for differences in rate of emergence and achieve the same plant density in WI and NI treatments: 151 plants/m^2^ for Soissons and Renan, 169 plants/m^2^ for Maxwell and Apache, and 164 plant/m^2^ for Caphorn. The density of plants was later controlled at three dates (November 14, December 4, and January 14), at which time there were no additional changes. Nitrogen fertilization followed the standard scheme with one dose at the tillering stage and one dose applied shortly before stem elongation. Crops were kept weed-free by herbicide application in March and later by regular survey and manual removing of weeds when necessary. Air temperature at 2 m, recorded from a nearby meteorological station, was used to compute the thermal time from plant emergence on an hourly basis, assuming a linear response to temperature with a base temperature of 0°C.

### Measurements at Crop Level: Ground Cover, PAIg, and Yield

Vertical ground cover GC (0°) was monitored weekly from ligulation of the fourth leaf of the main stem to approximately ear emergence (from February 8, 2013 to May 24, 2013). Six downward-looking photographs were taken at constant locations in each treatment. We used a NIKON camera D90, with a focal length of 50 mm which defines a field of view of 26.6°× 18.0°. Photographs were taken from ∼2.20 m height and each covered an area of 0.73 m^2^. Synchronously with vertical photographs, three oblique photographs with a direction of view at 57.5° from the vertical were acquired to monitor oblique ground cover GC (57.5°) and estimate the green plant area index PAIg (m^2^ leaf per m^2^ soil) as proposed by Baret et al. (2010). Oblique photographs each covered 2.03 m^2^. Photographs (0° and 57°) were processed using the SATVA software^1^ to determine the ground cover. Additionally, a direct measure of PAIg was made at flowering by dissecting 45 plants (three repetitions of 15 plants) per treatment and scanning all phytoelements.

The harvest was conducted mechanically on August 2 (∼2451°Cd after sowing) and grain yield (Mg/hectare) was measured on an area of 10 m^2^ per treatment, without replicates.

### Measurements at Plant Level: Tillering Dynamic, Rate of Leaf Emergence, and Geometry

**FIGURE 1:**
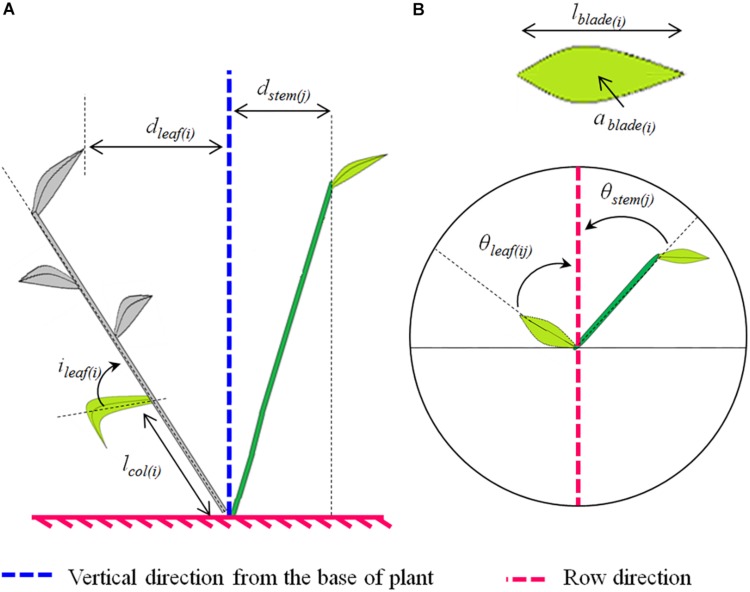
Scheme illustrating the plant architectural traits analyzed (**A**, side view; **B**, top view). The analyzed traits depended on the plant axis. For both main stems and tillers we analyzed: (i) the horizontal spread of the axis “*j*,” which represents the horizontal distance between the higher leaf collar on the axis and the plant base (*d*_*stem*(*j*)_); (ii) the horizontal spread of the leaf rank at rank “*i*” on the axis “*j*,” which represents the horizontal distance between the tip of the leaf and the plant base (*d*_*leaf*(*ij*)_); (iii) the azimuth angle of the leaf at rank “*i*” on the axis “*j*” which represents the angle between the projection of the midrib on the horizontal plane and the row direction (θ_*leaf*(*ij*)_); and (iv) the azimuth angle of the stem or pseudo-stem of the axis “*j*,” which represents the angle between the projection of the stem or pseudo-stem on the horizontal plane and the row direction (θ_*stem*(*j*)_). Additionally, the following traits were analyzed for the main stems only: (i) the final blade length of the leaf at rank “*i*”(*l*_*blade*(*i*)_); (ii) the mature blade area of the leaf at rank “*i*” (*a*_*blade*(*i*)_); (iii) the length between the collar of the leaf at rank “*i*” and the plant base (*l*_*col*(*i*)_); and (vi) the angle of insertion of the leaf at rank “*i*,” which represents the basal angle between the blade midrib and the bearing axis (*i*_*leaf*(*i*)_).

The measurements described below were performed for each treatment (cultivar^∗^interrow combination); the reader may refer to Figure 1, which depicts the measured variables.

The number of axes per plant was measured five times during the crop cycle, from the emergence of leaf 5 of the main stem to flowering. Measurements were conducted on 10–50 plants that were collected in the field and brought to the laboratory where axes having at least one emerged green leaf were recorded.

The number and exposed length of individual leaf blades on the main stems were measured six to seven times during the crop cycle on 10 tagged plants to characterize the leaf stage as described in Abichou et al. (2018). The rate of leaf emergence was estimated by adjusting a linear model between leaf stage and thermal time.

The length of the pseudo stem *l*_*col*(1)_ (from the base of the plant up to the highest collar) and the length of the shoot *l*_*apex*_ (from the base of plant to the summit of the main stem apex) were measured with a ruler on 30 tagged plants at 870°Cd after emergence (leaf stage ∼8.5).

The final dimensions of upper main stem phytomers were measured on 30 tagged plants after flag leaf ligulation (1278°Cd) and after completion of stem extension (1565.5°Cd). The number of elongated internodes – having lengths ≥0.5 cm – was recorded and the following traits were calculated for each leaf rank (leaves are numbered basipetally, with *i* = 1 for the flag leaf): (i) the mean blade length, maximum width, and area (*l*_*blade*(*i*),_ *w*_*blade*(*i*),_ *a*_*blade*(*i*)_) and (ii) the mean length from the base of the plant to each leaf collar (*l*_*col*(*i*)_). The lengths were measured with a ruler, then plants were dissected and blade images were obtained with a flat-bed scanner and were processed using the program Lamina2Shape (Dornbusch and Andrieu, 2010) to measure their area and green fraction.

To follow the dynamic of the insertion angle of leaf blades *i*_*leaf*(*i*)_, destructive measurements were carried out on 30 tagged main stems, at seven dates from the start of stem elongation to full senescence. Photographs of stems with their attached leaves were analyzed with the POPCORN software (Weiss and de Solan (see footnote 1), to register the coordinates of points along the stem and the leaf midrib. An R routine was developed to compute the angle of insertion *i*_*leaf(i)*_ for each leaf rank, considering only leaves having at least 20% green area.

**FIGURE 2:**
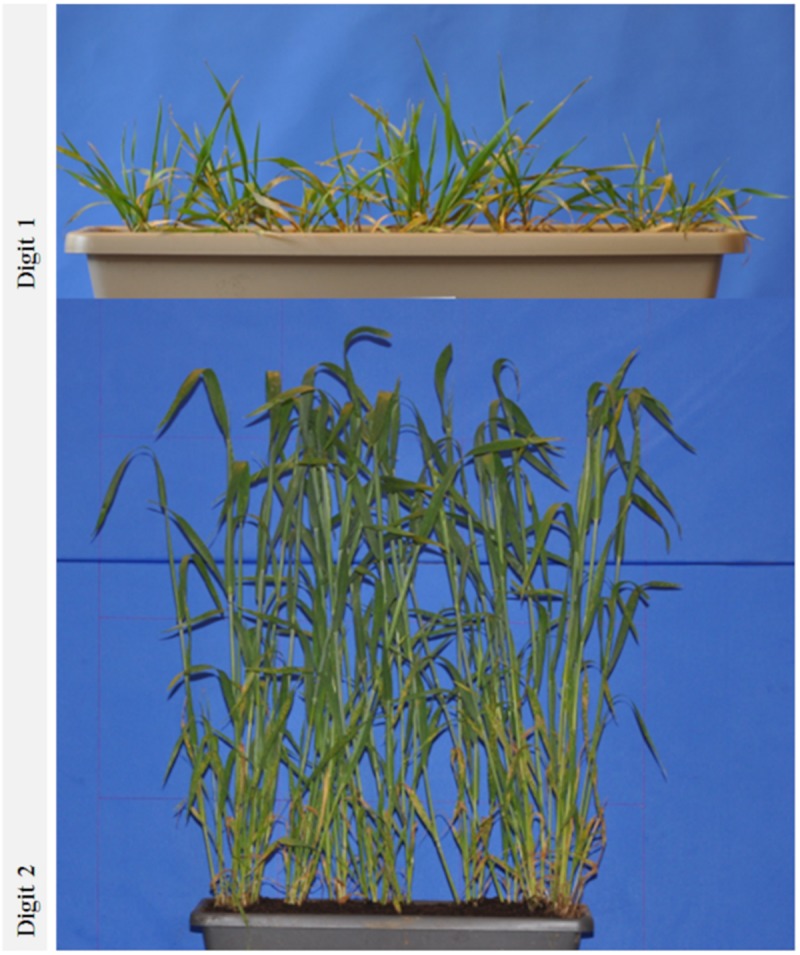
Photographs of segments of rows analyzed. Rows were collected from the field at 722°Cd (top) and 1272°Cd (bottom) and transplanted in pots to be digitalized in the laboratory. The treatment shown here is Renan-NI.

Comprehensive characterizations of 3D geometry of plants were conducted at two stages: just before stem extension (722°Cd; leaf stage∼6.9), and around ear emergence (1272°Cd) (see Figure 2). In each treatment, two segments of rows (40 cm length) were collected, including the soil and the roots to a depth of ∼15 cm and each comprising ∼15 plants in NI treatments and ∼30 plants in WI treatments. Row segments were put intact into pots and brought to the laboratory, with great care to preserve the canopy structure as it was in the field. Segments were photographed (Supplementary Figures S3, S4), then the coordinates of points along leaves midribs and stems were recorded with a magnetic 3D digitizer (3Space Fastrak, Polhemus Inc., Colchester, VT, United States) using the 3A software (Sinoquet et al., 1997; Adam et al., 1999). For each treatment, the process yielded the 3D skeleton of all axes and leaves of plants in the row segments in the position they were in the field.

From these 3D skeletons, the following traits were calculated (see Figure 1):

1. *d*_*stem*_: the mean horizontal spread between the top of the stem and the base of the plant, taking into account all plants axes. The horizontal spread of an axis is the horizontal distance between the collar of the upper leaf and the plant base;
2. θ_*stem*_: the mean azimuth angle of stems, taking into account all plants axes. The azimuth angle of an axis represents the angle between the projection of this axis on the horizontal plane and the row direction;
3. *d*_*leaf*_: the mean horizontal spread of leaves, taking into account all plants leaves. The spread of a leaf is the horizontal distance between the tip of this leaf and the plant base;
4. θ_*leaf*_: the mean azimuth angle of leaves, taking into account all plants leaves. The azimuth angle of a leaf is the angle between the projection of the leaf midrib on the horizontal plane and the row direction.

For the second date of digitalization, these traits (*d*_*stem*_, *d*_*leaf*,_ θ_*stem*_, θ_*leaf*_) were calculated considering only well-developed axes (i.e., those ≥10 cm in height).

3D skeletons were also used to calculate the mean inclination angle of blade surfaces (α), a trait widely used to characterize the canopy structure in light interception models. For this, the 3D skeletons, together with information on the 2D leaf shapes obtained from the leaf scans, were used to simulate the architecture of the canopy as a 3D mesh composed of triangles (Pradal et al., 2009). The inclination angle of a triangle is the angle between the direction perpendicular to the triangle and the vertical direction (only considering the range between 0 and 90°). The mean inclination angle (α) was calculated by averaging the inclination angles of all triangles representing blade surfaces, weighted by their corresponding areas.

### Statistical Analysis

Mean values and 95% confidence intervals were calculated for the majority of measured traits. The significance of the difference in the mean value of a trait between NI and WI treatments was tested using either the non-parametric test of Mann and Whitney (for samples < 30) or the Student’s *t* test (for samples ≥ 30) (Georgin and Gouet, 2000), using R software. The results of the detailed analysis of the measured variables are shown in (Supplementary Table S1). For grain yield, there were no replicates, so confidence intervals could not be calculated.

## Results

### Tillering and Yield

**FIGURE 3:**
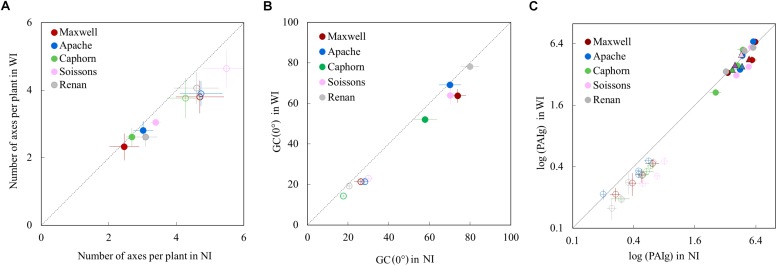
The number of axes per plant **(A)**, the vertical ground cover GC(0°) **(B)**, and the green plant area index PAIg **(C)**, in WI treatments vs. NI treatments. Each color is associated with a cultivar, as indicated in the legend. Error bars represent the 95% confidence intervals of mean estimates and the dotted line represents the line 1:1. In **(A)**, each symbol represents the mean value of the number of axes measured for 20–30 plants per treatment. Empty symbols are for end of tillering (∼870°Cd) and full symbols are for flowering. In **(B)**, each symbol shows the mean value of GC(0) from analyzing six photographs covering each an area of 0.73 m^2^. Empty symbols represent measurements at 682°Cd (LS = 5.6) and full symbols represent measurements at 1565°Cd (after flag ligulation). In **(C)**, circles show the PAIg estimated from oblique photographs: estimates at early stages are shown with empty circles (520°Cd, 532°Cd, 657°Cd, and 683°Cd) and estimates at later stages are shown with full circles (952°Cd, 1222°Cd, and 1555°Cd). Each circle shows the mean of values calculated from analyzing three photographs covering each area of 2.03 m^2^. Triangles represent the PAIg measured at flowering (1575°Cd) from scans of leaves and stems.

Increasing row spacing resulted in a lower rate of tiller emergence and a lower value for the maximum number of emerged tillers. At the end of tillering, the number of axes in WI treatments was below that in NI treatments, with a difference ranging from 12 to 19% depending on the cultivar (Figure 3A). We found that a lower rate of tiller emergence occurred in WI despite a higher rate of leaf emergence and a higher final leaf number, compared to NI (see below). Additionally, a reduction of tillering was observed in Caphorn and Soissons from an early stage (LS∼5), when mutual shading was still negligible (Supplementary Figure S1A). During the tiller mortality phase, the differences between WI and NI decreased and finally the number of ears in WI treatments ranged by 3% (Caphorn) to 15% (Renan) below those in NI treatments (Figure 3A). Despite the lower ear density under WI, there was no overall trend for grain yield. Differences calculated for each cultivar were low (Table 1), except for Apache (−5% in WI); our protocol did not allow us to calculate confidence intervals.

**TABLE 1:** The mean values of grain yield and ear number per plant for the 10 treatments.

	**Maxwell**		**Apache**		**Caphorn**		**Soissons**		**Renan**	
	**NI**	**WI**	**NI**	**WI**	**NI**	**WI**	**NI**	**WI**	**NI**	**WI**
Grain yield (Mg/ha)	5.4	5.51	5.36	5.09	4.54	4.67	4.34	4.38	3.84	3.81
Ear number	2.5	2.3	3	2.8	2.7	2.6	3.4	3	3.1	2.8

### Ground Cover and PAIg

The vertical ground cover GC (0°) was generally lower in WI compared to NI, as expected from the sowing configuration and the reduction of tillering. For Apache and Renan, differences between WI and NI were not apparent at the end of cycle (Figure 3B), meaning that these cultivars fully compensated for the change of sowing configuration. For the other cultivars, ground cover was consistently lower in WI treatments (the dynamics of ground cover are shown in Supplementary Figure S1B).

Increasing row spacing also reduced the plant green area index (PAIg) at the early stages of the crop cycle with a mean difference of 31.3 ± 4.8% (Figure 3C). This reduction is consistent with the lower tillering in WI treatments. At flowering, no significant differences in PAIg were observed between WI and NI treatments, despite the slightly lower ear density in WI. This means that the larger size of upper leaves blades in WI treatments (reported below) fully compensated for the difference in the number of ear-bearing shoots.

### Plant Development

**FIGURE 4:**
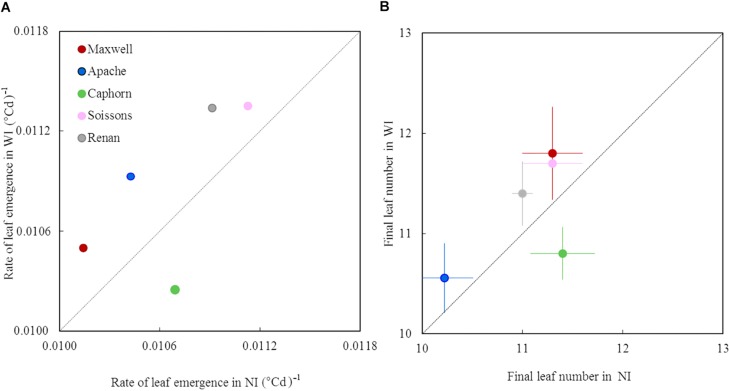
The rate of leaf emergence and the final leaf number of the main stem in WI treatments vs. NI treatments. Each color is associated with a cultivar, as indicated in the legend. The value of the rate of leaf emergence **(A)** was estimated from a linear model adjusted on the mean estimates of leaf stage. The data for final leaf number **(B)** are the mean values measured on 10 tagged plants, with error bars representing 95% confidence intervals of mean estimates. The dotted line represents the line 1:1.

Increasing row spacing resulted in a higher rate of leaf emergence for all cultivars except Caphorn (Figure 4A). Apache, Renan, Maxwell, and Soissons showed increases ranging from 2 to 5%, while Caphorn showed a decrease of 4% under WI treatments. The changes in leaf emergence rate were associated with parallel changes in the total number of leaves produced by the main stem: +0.4 leaves for Renan and Soissons (*p* = 0.04 and 0.08, respectively), +0.3 and +0.5 leaves for Apache and Maxwell (*p* = 0.17 and 0.21, respectively), and −0.6 leaves for Caphorn (*p* = 0.01) (Figure 4B). The parallel changes in the rate of leaf emergence and the total number of leaves produced resulted in the ligulation of the flag leaves occurring synchronously in NI and WI (see Supplementary Figure S1C).

**FIGURE 5:**
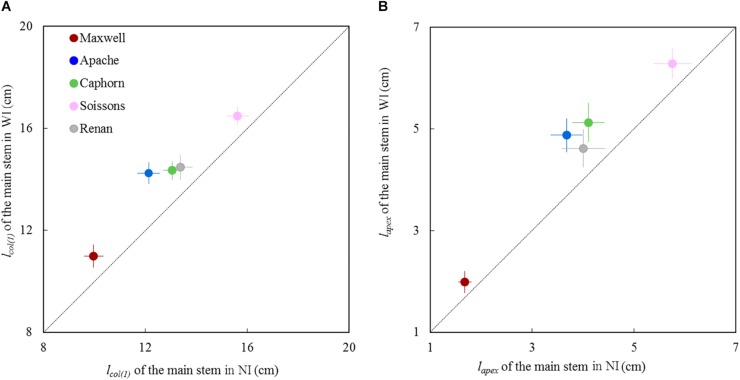
Length of the main stem in WI treatments vs. NI treatments at the start of stem extension (870°Cd). **(A)** shows *l*_*col*(1)_, which represents the length from the base of the plant up to the highest collar and figure **(B)** shows *l*_*apex*_, the length from the base of plant to the summit of the apex. Each symbol represents the mean of values measured on 30 stems. Each color is associated with a cultivar, as indicated in the legend. Error bars represent 95% confidence intervals of mean estimates. The dotted line represents the line 1:1.

For all cultivars, the length of the pseudo stem *l*_*col*(1)_ and the length of the shoot *l*_*apex*_ measured around the start of stem elongation (875°Cd; LS∼8.5) were significantly higher in WI compared to NI (Figure 5). The higher values of *l*_*col*(1)_ and *l*_*apex*_ suggest either a faster progress toward the reproductive stage for plants grown under WI treatments or an enhanced sheath and internode extension in response to competition signals.

### Dimensions of Mature Plant Organs

**FIGURE 6:**
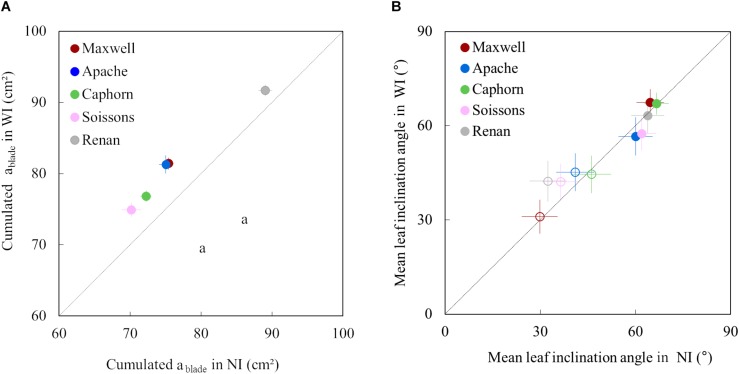
The cumulative area of the four upper blades of the main stem **(A)** and the mean leaf inclination angle α **(B)** in WI treatments vs. NI treatments. Each color is associated with a cultivar, as indicated in the legend. The dotted line represents the line 1:1. In **(A)**, symbols represent the mean of values measured on 45 plants per treatment; error bars mark the 95% confidence intervals of the mean estimates. In **(B)**, empty symbols represent measurements at the start of stem extension (722°Cd) while full symbols represent measurements close to ear emergence (1272°Cd); error bars mark the quarter of standard deviation of mean estimates of all triangles representing leaves (to make the figure easier to read).

Increasing row spacing resulted in an increase of the blade area of upper leaves for all cultivars. The cumulative area of the fourth upper blades was 3–8% higher in WI compared to NI (Figure 6A). This resulted from an increase in both length and width of the upper leaves.

After completion of stem extension, the distances from the base of the plant to the collar of the upper leaves *l*_*col*(*i*)_ were not impacted by row spacing. This suggests that the vertical distribution of upper leaf insertions was unaffected despite the changes in final leaf number (see Supplementary Figure S2). There was, however, an increase (+0.2 to +0.5) in the number of elongated internodes for some cultivars (Caphorn, Soissons, and Maxwell) in WI compared to NI, which, together with the higher values of *l*_*apex*_ mentioned above, indicates that stem extension was transitorily enhanced in WI in the early stages.

### Leaf Angle

**FIGURE 7:**
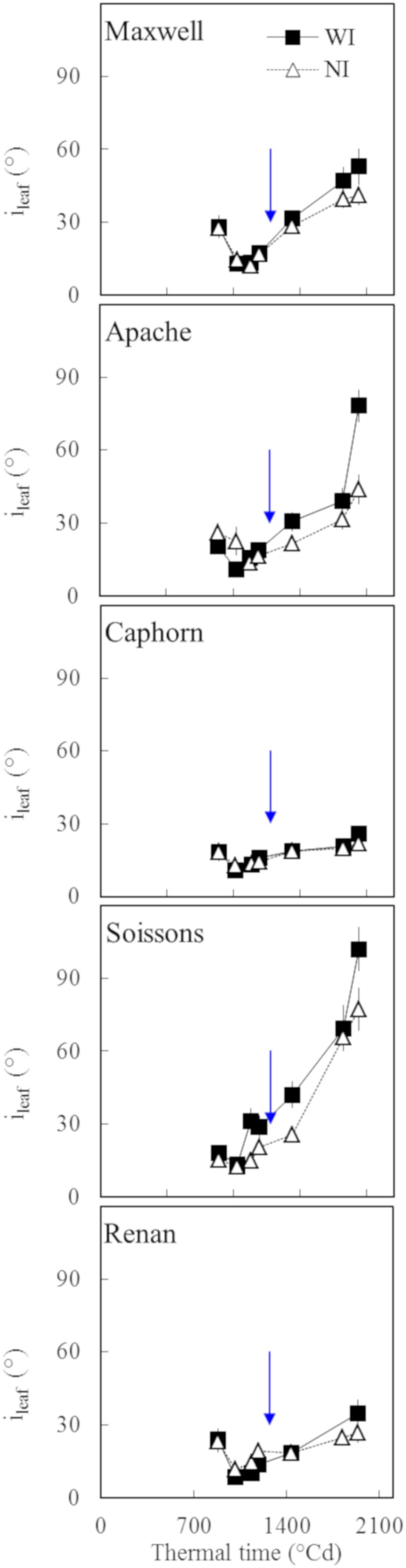
The insertion angle (*i*_*leaf*_) averaged over the four upper leaves of the main stem vs. the thermal time from plant emergence. Each symbol represents the mean of values measured on 30 plants. Squares and solid lines represent the WI treatment, while triangles and dotted lines represent the NI treatment. Only leaf blades, having at least 20% green area, were taken into account. Error bars mark 95% confidence intervals of the mean estimates. The arrow indicates the second date of digitalization (1272°Cd), which is close to ear emergence date.

Two indicators of leaf structure were used to evaluate the effect of increasing row spacing: (a) the average inclination angle of all canopy leaves α (Figure 6B) and (b) the mean insertion angle *i*_*leaf*_, calculated by averaging *i*_*leaf*(*i*)_ for the four upper leaves of the main stem (Figure 7).

For all cultivars and row spacing treatments, α was lower at the early stage (722°Cd) than around ear emergence (1272°Cd). This result was somewhat expected as planar leaves increase light interception in a sparse canopy while inclined leaves allow light penetration through a dense canopy. Increasing row spacing had little impacts on α, but some trends were observed for the different cultivars. For Maxwell and Caphorn, the α values were similar in NI and WI, suggesting no changes in leaf posture. Other cultivars showed a trend for the impact of interrow distance on α. At 722°Cd, the leaves of cultivars Renan and Soissons were slightly more erect in WI compared to NI, which could be interpreted as an enhanced shade avoidance response. Oppositely, at 1272°Cd, Apache and Soissons showed a slight trend of a lower inclination angle in WI, consistent with a lower competitive pressure. Differences in α between treatments were small, but it is worth mentioning that differences in α were small also when comparing between cultivars having different statures. For example, the leaf inclination angle of Renan-NI (dropping stature) and Caphorn-NI (erect stature) were 19.8° and 17.4°, respectively. This means that the widely used mean leaf inclination angle has a low sensitivity to the differences in stature between cultivars.

We further analyzed the changes in plant stature by investigating the mean insertion angle of the four uppers leaves of the stem *i*_*leaf*_ (Figure 7). Marked differences were observed depending on the cultivar: Apache, Soissons, and Maxwell showed a significantly higher *i*_*leaf*_ in WI compared to NI, especially after ear emergence, which could be interpreted as an active response to explore more of the free space. For Maxwell, this behavior started later than it did for Apache and Soissons, so that no difference existed at the time when α was estimated. A detailed analysis of the behavior for each leaf ranks showed that the higher *i*_*leaf*_ in WI compared to NI resulted from differences in the dynamics of leaf insertion angle with leaf age, but also from the differences in plant development (accelerated growth, slowed senescence, and higher final leaf number in WI). The difference in *i*_*leaf*_ between WI and NI increased with time and it is likely that α followed the same pattern. On the other hand, Caphorn and Renan did not show any impact of interrow on *i*_*leaf*_. Additionally, despite the noticeable difference in their stature (erect for Caphorn, sagging for Renan), they had similar values of *i*_*leaf*_ and were the lowest among the examined cultivars. This suggests that the sagging stature of Renan is not related to a high value of *i*_*leaf*_, but rather in the curvature of blades.

### Horizontal Spread and Azimuth Orientation of Leaves and Stems

**FIGURE 8:**
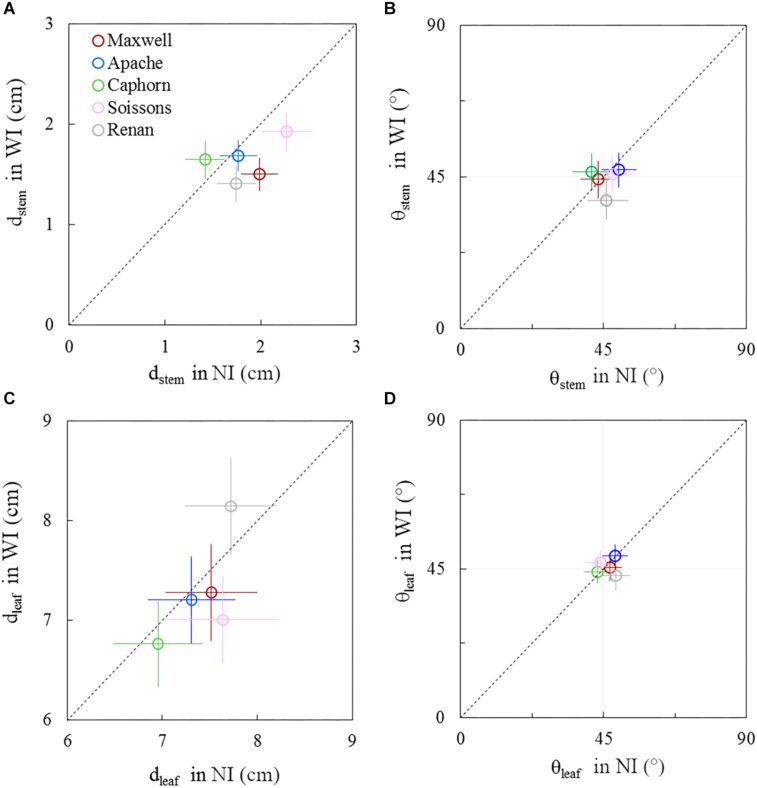
Horizontal spacing and azimuth orientation of stems and leaves in WI treatments vs. NI treatments, at 722°Cd. The stem spread (*d*_*stem*_; **A**), leaf spread (*d*_*leaf*_; **C**), stem azimuth angle (θ_*stem*_; **B**), and leaf azimuth angle (θ_*leaf*_; **D**). Symbols represent the mean values taking into account all axes, for 30–60 plants per treatment. Error bars represent 95% confidence intervals of the mean estimates. The dotted line represents the line 1:1.

**FIGURE 9:**
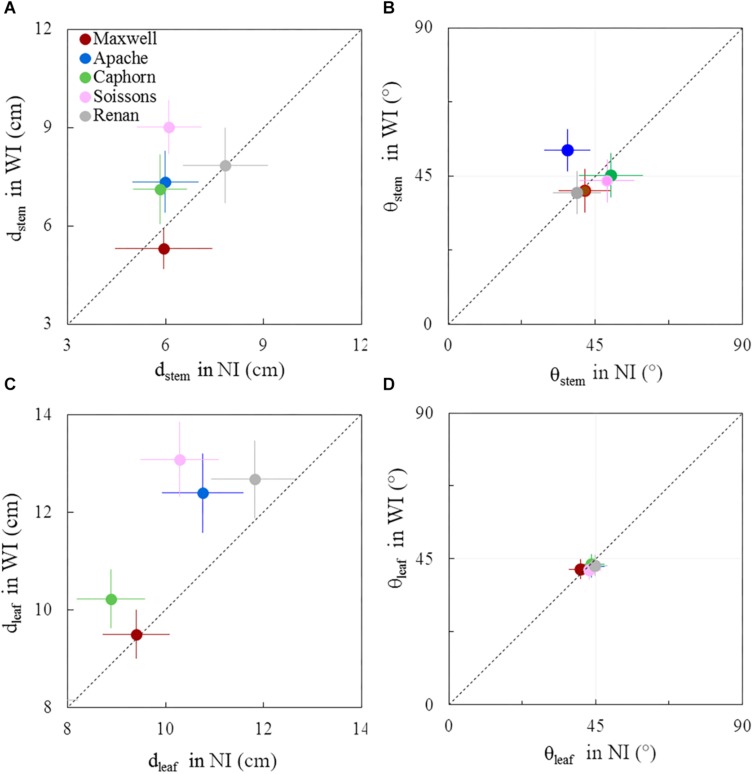
Horizontal spacing and azimuth orientation of stems and leaves in WI treatments vs. NI treatments, at 1272°Cd. The stem spread (*d*_*stem*_; **A**), leaf spread (*d*_*leaf*_; **C**), stem azimuth angle (θ_*stem*_; **B**), and leaf azimuth angle (θ_*leaf*_; **D**). Symbols represent the mean values taking into account only well-developed axes, with h_*col*(1)_ > 10 cm, for 30–60 plants per treatment. Error bars represent 95% confidence intervals of the mean estimates. The dotted line represents the line 1:1.

The geometry of plant axes and leaves showed moderate differences between row spacing treatments. These responses changed during the crop cycle and differed among cultivars. These geometrical responses concern the horizontal spreading and azimuth orientation of leaves and stems (*d*_*stem*_, *d*_*leaf*_, θ_*stem*_, θ_*leaf*_). A comparison between WI and NI treatments is presented in Figures 8, 9, respectively, for 722°Cd and 1272°Cd (photographs are shown in Supplementary Figures S3, S4).

Before the start of stem elongation, plants had a lower *d*_*stem*_ in WI treatments compared to NI, especially for Maxwell, Renan, and Soissons, for which differences ranged from 15 to 25% (Figure 8A). At this stage the pseudo-stems were longer in WI treatments, with higher *l*_*col*(1)_ as presented previously, such that the lower *d*_*stem*_ means that the pseudo-stems were more erect in WI. For Caphorn and Apache, no significant difference in *d*_*stem*_ was observed between WI and NI treatments. Concerning the horizontal spreading of leaves *d*_*leaf*_, only Soissons showed a significant decrease by 8%, meaning that leaves were more erect as observed for pseudo-stems. Renan showed an opposite trend but it was not significant. In the other hand, no trend was observed on the azimuth angle of the pseudo-stems θ_*stem*_ and leaves θ_*leaf*_ for all cultivars, except Renan, meaning that for the other cultivars there was no preferential orientation of plant axes and leaves relative to row direction (Figures 8B–D). Oppositely, Renan, which had a higher *d*_*leaf*_, showed an unexpected response with slightly lower θ_*stem*_ and θ_*leaf*_ in WI compared to that in NI, which suggested that both stems and leaves were orientated toward the neighbor plants in the same row.

Around ear emergence, *d*_*stem*_ differed markedly between cultivars (*p* < 0.0001) and row spacing (*p* = 0.0067), ranging from 5.3 cm for Maxwell-WI to 9 cm for Soissons-WI (Figure 9A). The general trend was opposite of that observed at the start of stem extension, as wide interrows resulted in an increase of *d*_*stem*_ by 48% for Soissons (*p* < 0.001) and by 23% for Apache (*p* < 0.1) and Caphorn (ns), while other cultivars showed no marked change. Since plant height was not affected by row spacing (see section above), the difference in *d*_*stem*_ indicates that plant stems spread more horizontally in WI compared to NI. The wider spread of axes of Soissons and Caphorn in the WI treatment was not associated with a specific orientation toward the interrow space (Figure 9B); by contrast, Apache showed a significant orientation of axes toward the free space. Consistent with the wider spread of axes, the distribution of leaf tips showed a higher scatter in WI treatments, with a significant increase of *d*_*leaf*_ by 27, 15, and 14, respectively, for Soissons, Caphorn, and Apache, but without any trend toward an interrow orientation in WI treatments, as the mean leaf azimuth θ_*leaf*_ was 45° in all cases (Figure 9D).

## Discussion

At the early stages, most of the reactions of plants grown under wide row spacing mimicked the shade avoidance responses usually observed for plants grown at higher density. As summarized in Table 2, in their first stage, plants in WI showed a reduction in the number of tillers for all cultivars, with a trend of having more erect and grouped pseudo-stems. This association of changes in tiller number and angle is typical of grass responses to density changes as reported by Gibson et al. (1992). Furthermore, plants in WI showed a trend of having an earlier stem extension, which is also an expected response to higher density. We observed in WI a slightly faster rate of leaf development for all cultivars (except Caphorn), together with a slightly higher final leaf number, while the date of flag leaf ligulation was unchanged. Correlated differences in the rate of leaf production and leaf number without changes in flag leaf ligulation have been reported when comparing density treatments (Baccar, 2011) and when analyzing plant-to-plant variability within the same field (Abichou et al., 2018). However, an increase in leaf number is usually associated with a lower plant density, so the slightly higher leaf number in WI conflicts with the other changes that denote a higher perception of competition in this treatment at the early stages. The exact processes and timing of the determination of the final leaf number are not fully understood and we hypothesize that they may have been impacted by the change from a perception of higher density to a perception of lower density in WI, which may have occurred before leaf stage 7, as discussed below. The higher perception of competition in WI suggests that the distance to the closest neighbors plays a specific role in the perception of competition. Differences in tillering between treatments already existed at LS∼5.27 ± 0.36 when ground cover was around 15%, for which resources were likely not limiting. Such behavior may have been triggered by phytochrome, mechanosensing, or volatile signaling as reported for other species (Ballaré, 1999; de Wit et al., 2012). Our work does not allow us to distinguish between these possible triggers, but at the stage when the first responses were observed, there were many contacts between neighboring plants within the same row in the WI treatments; so responses to touch signals would be a simple explanation for the exacerbated perception of competition in the WI. In this early stage, plants did not show any tendency to specifically avoid their closest neighbors in the row or to prospect the interrow space, suggesting they lacked the ability to directionally respond to their anisotropic environment. More surprising, Renan showed a slight trend to orient axes and leaves toward the closest neighbors within a row.

**TABLE 2:** Summary of the effect of increasing row spacing on architecture variables.

**Traits**				**Sign of response**		
		**Maxwell**	**Apache**	**Caphorn**	**Soissons**	**Renan**
Rate of leaf emergence on MS		**+**	**+**	**−**	**+**	**+**
Final leaf number of MS		**+**	**+**	**−**	**+**	**+**
Number of elongated internodes		**+**	**−**	**+**	**+**	**−**
Maximum number of axes		**−**	**−**	**−**		
Ear number		**−**	**−**	**−**	**−**	**−**
Grain yield		**+**	**−**	**+**	**+**	**−**
PAIg around flowering (1565°Cd)		**−**	**−**	**−**	**−**	**+**
Blade area of flag leaf		**+**	**+**	**+**	**+**	**+**
Blade area of second leaf		**+**	**+**	**+**	**+**	**+**
Blade area of third leaf		**+**	**+**	**+**	**+**	**+**
Blade area of fourth leaf		**+**	**+**	**+**	**−**	**+**
*l*_*apex*_ of MS at 870°CD		**+**	**+**	**+**	**+**	**+**
*l*_*col*(1)_ of MS at 870°CD		**+**	**+**	**+**	**+**	**+**
*l*_*col*(1)_ of MS at 2287°CD		**+**	**+**	**+**	**+**	**+**
*i*_*leaf*_ of the fourth upper leaves at 888°Cd		**−**	**−**	**−**	**+**	**+**
	at 1015°Cd	**−**	**−**	**−**	**+**	**−**
	at 1139°Cd	**+**	**+**	**−**	**−**	**−**
	at 1191°Cd	**−**	**+**	**+**	**−**	**−**
	at 1439°Cd	**+**	**+**	**−**	**+**	**+**
	at 1820°Cd	**+**	**+**	**+**	**−**	na
	at 1940°Cd	**+**	**+**	**+**	**+**	**+**
*d*_*stem*_	at 722°Cd	**−**	**−**	**+**	**−**	**−**
θ_*stem*_	at 722°Cd	**+**	**−**	**+**	**−**	**−**
*d*_*leaf*_	at 722°Cd	**−**	**−**	**−**	**−**	**+**
θ_*leaf*_	at 722°Cd	**−**	**+**	**+**	**+**	**−**
*d*_*stem*_	at 1272°Cd	**−**	**+**	**+**	**+**	**+**
θ_*stem*_	at 1272°Cd	**−**	**+**	**−**	**−**	**+**
*d*_*leaf*_	at 1272°Cd	**+**	**+**	**+**	**+**	**+**
θ_*leaf*_	at 1272°Cd	**+**	**−**	**−**	**−**	**−**

*The (+) symbol refers to an increased effect and the (**−**) symbol refers to a decreased effect. A gray background means that the effect was significant (p < 0.1) and white background means that effect was not significant (except for the rate of leaf emergence and grain yield for which statistical tests were not possible).*

After the start of stem elongation, plants grown with wide interrows showed a change in behavior, with a lower regression of tillers and a significant increase in the area of the fourth upper blades, which compensated for the reduction of tillering. This behavior seems to reflect a higher availability of resources per axis and the mature architecture of flowering axes mimicked those of plants grown at lower density. Such a trend was also reported earlier by Hiltbrunner et al. (2005). Leaf 8 was already significantly larger in WI compared to NI, implying that the changes already existed during the development of leaf 8; that is around leaf stage 7 or earlier. Besides, marked geometrical adaptations according to cultivars were observed: (i) stems grew more obliquely for Soissons, Apache, and Caphorn (Figure 9A), (ii) leaves had a higher insertion angle in Soissons, Apache, and Maxwell, leading to a more planophilic stature, especially after ear emergence (Figure 7), (iii) Soissons, Renan, Maxwell, and Caphorn showed no trend for the azimuth angle of leaves and stems, meaning that plants did not orient themselves toward the free space between rows; oppositely, Apache preferentially oriented the axes toward the free space between rows (Figure 9B). Interestingly, the azimuth angle of leaves was not affected (Figure 9D), contrary to what is widely reported for maize, in which the plants tend to align their leaves perpendicularly to the row direction to avoid their neighbors in the row (Drouet and Moulia, 1997; Maddonni et al., 2002).

Overall, our work demonstrated marked changes in tillering behavior, leaf size, and geometric display of leaves and axes in WI compared to NI. This was accompanied by changes in phyllochron and final leaf number. There were genotypic differences for the aerial traits responding to interrow spacing but differences in yield between NI and WI were small. Our experimental design did not allow us to estimate their statistical significance. Furthermore, other traits that we did not quantify, such as shoot/root ratio, leaf green duration, or harvest index, may be important here. The general picture that emerges is that in WI there was an enhanced perception of competition in the first stage of growth, followed by a response to higher level of resources in the later stage of growth, whereas, with the exception of Apache, there was no sign of a specific avoidance of the region of denser canopy or preferential foraging of the gap areas. The behavior of Apache suggests that genetic variability may exist in wheat for the ability to react to crop heterogeneity by foraging gaps. In maize, Maddonni et al. (2001) distinguished cultivars having “plastic” or “rigid” behavior according to their ability to re-orientate their leaves toward interrows. A directional response may provide competitive advantage not only in the case of large interrows, but also in other situations such as competition with weeds or cultures grown in association.

The traits that differed in response to interrow distance evolved dynamically along the whole plant cycle. Accordingly, their impact can hardly be estimated from measurements at a few dates, but rather could be investigated by simulation with dynamic 3D plant models (Vos et al., 2010). Our work provides data that could be used in structural, data-driven 3D models such as Adel-Wheat (Fournier et al., 2003), which allow calculation of plant–environment interactions based on realistic 3D dynamic structures. This would help in defining ideotypes for light capture, and to investigate other consequences of the changes in sowing design and plant architecture, such as the perception of light quality (Chelle et al., 2007), propagation of pathogens (Robert et al., 2018), interception of pesticides (Dorr et al., 2008), or the formation of phenotyping signals (Liu et al., 2017).

Our results also provide hints for the development of functional (process-driven) models. The realistic representation of plant architecture in functional-structural plant models (FSPM) has opened up new possibilities to simulate the behavior of plants in heterogeneous environments, but many questions remain about the signals perceived, how they are integrated over time and space, and what are the quantitative reactions to the signals (Bongers et al., 2014). Progress is needed not only to generate better mechanistic models but also to identify simple proxies that can be used without complex computations. Our work revealed two original results. First, responses appeared to be related to the distance to the closest neighbors rather than to the average foliage density in the plant neighborhood. Second, the responses affected the spreading behavior, but for most cultivars, the responses were not directional.

These observations correspond to a single year-site experiment and involved intrusive methods to characterize the 3D architecture. High throughput phenotyping with 2D and LiDAR imagery (Liu et al., 2017) allows investigating in field conditions and without any contact with the plants, a large number of cultivar traits such as position of individual ears and leaf orientation, which in the present study were measured by digitization. In this context, manipulating interrow distance is a convenient way to create reproducible representations of spatial heterogeneity. If confirmed, the behavior reported here would be an important element to help model the response of wheat plants not only to interrow spacing, but also to a large range of situations of heterogeneity.

## Conclusion

We found that wheat plants responded to changes in interrow distance by altering tillering, leaf size, and leaf and axes spreading in a manner that corresponded to an altered perception of competition along the cycle. Among the five cultivars tested, only one showed a small tendency to orientate axes so as to forage preferentially the interrow space. These results help to better understand and model the response of wheat plants to specific management techniques and more generally to spatial heterogeneities.

## Data Availability

All datasets for this study are included in the manuscript and the Supplementary Files.

## Author Contributions

MA performed the experiments, analyzed the data, and wrote the initial manuscript. BA conceived the study and revised the manuscript. BdS helped to conceive the study and obtained the funding. All authors read and approved the final manuscript.

## Conflict of Interest Statement

The authors declare that the research was conducted in the absence of any commercial or financial relationships that could be construed as a potential conflict of interest.

## Footnotes

1 https://www6.paca.inra.fr/emmah/Production-Documentation/Outils-et-modeles
