# Supplementary material for: Architectural Response of Wheat Cultivars to Row Spacing Reveals Altered Perception of Plant Density
Source: Front Plant Sci. 2019 Aug 7;10:999. doi: 10.3389/fpls.2019.00999 (PMC6692534; doi:10.3389/fpls.2019.00999)
Supplement: Supplementary file 1 [file Data_Sheet_1.docx]

Supplementary Material

**Architectural Response of Wheat Cultivars to Row Spacing Reveals Altered Perception of Plant Density**

**Mariem Abichou, Benoit de Solan, Bruno Andrieu***

*** Correspondence:** Bruno Andrieu : [bruno.andrieu@inra.fr](mailto:bruno.andrieu@inra.fr)

**
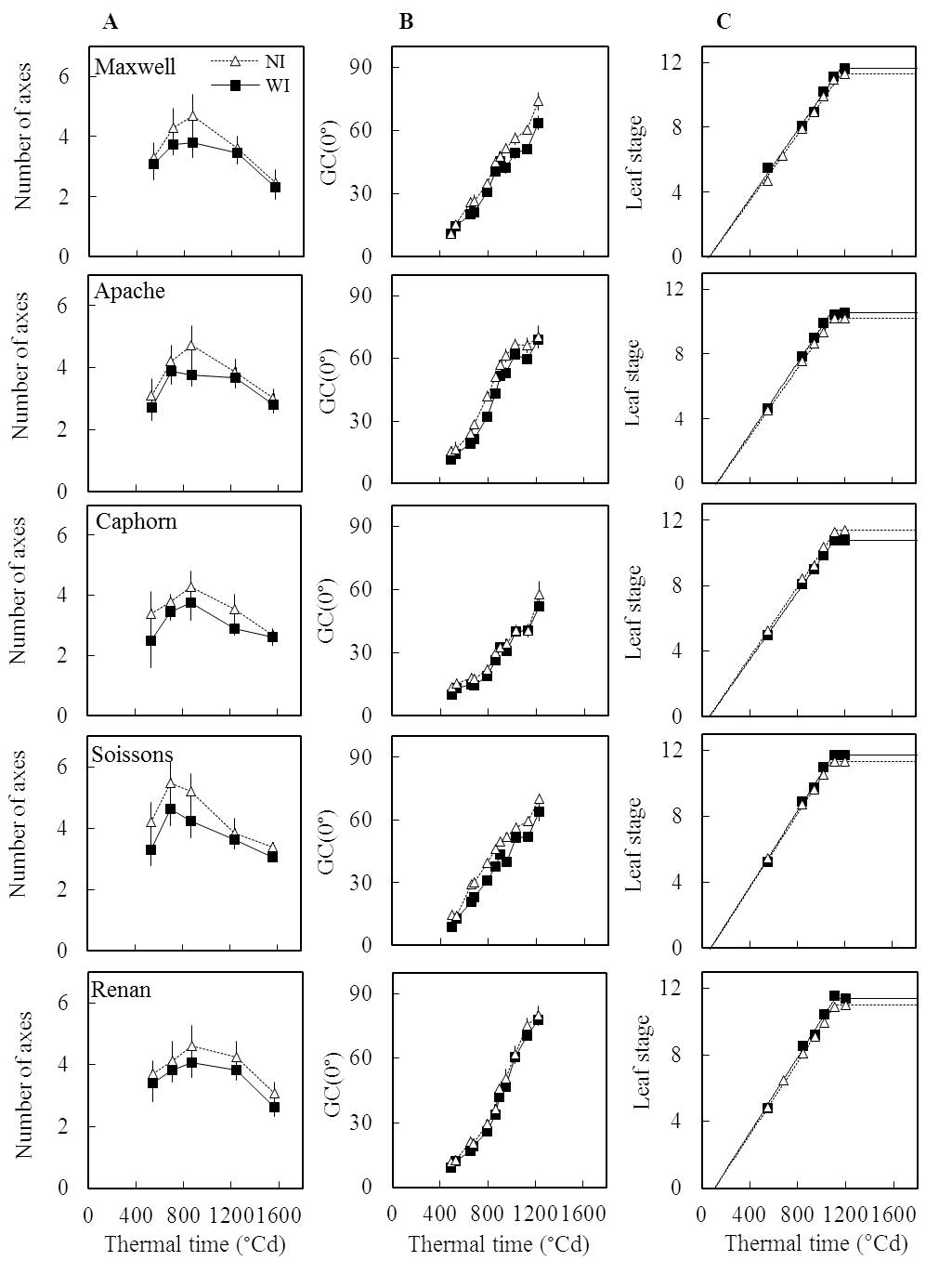
**

**Supplementary Figure S1 |** Dynamics of architecture traits vs thermal time after emergence, for winter wheat cultivars Maxwell, Apache, Caphorn, Soissons and Renan in NI (empty triangles) and WI (filled squares) treatments : number of active axes per plant (A), ground cover (B), leaf stage (C). Vertical bars mark the 95% confidence intervals of the mean estimates.


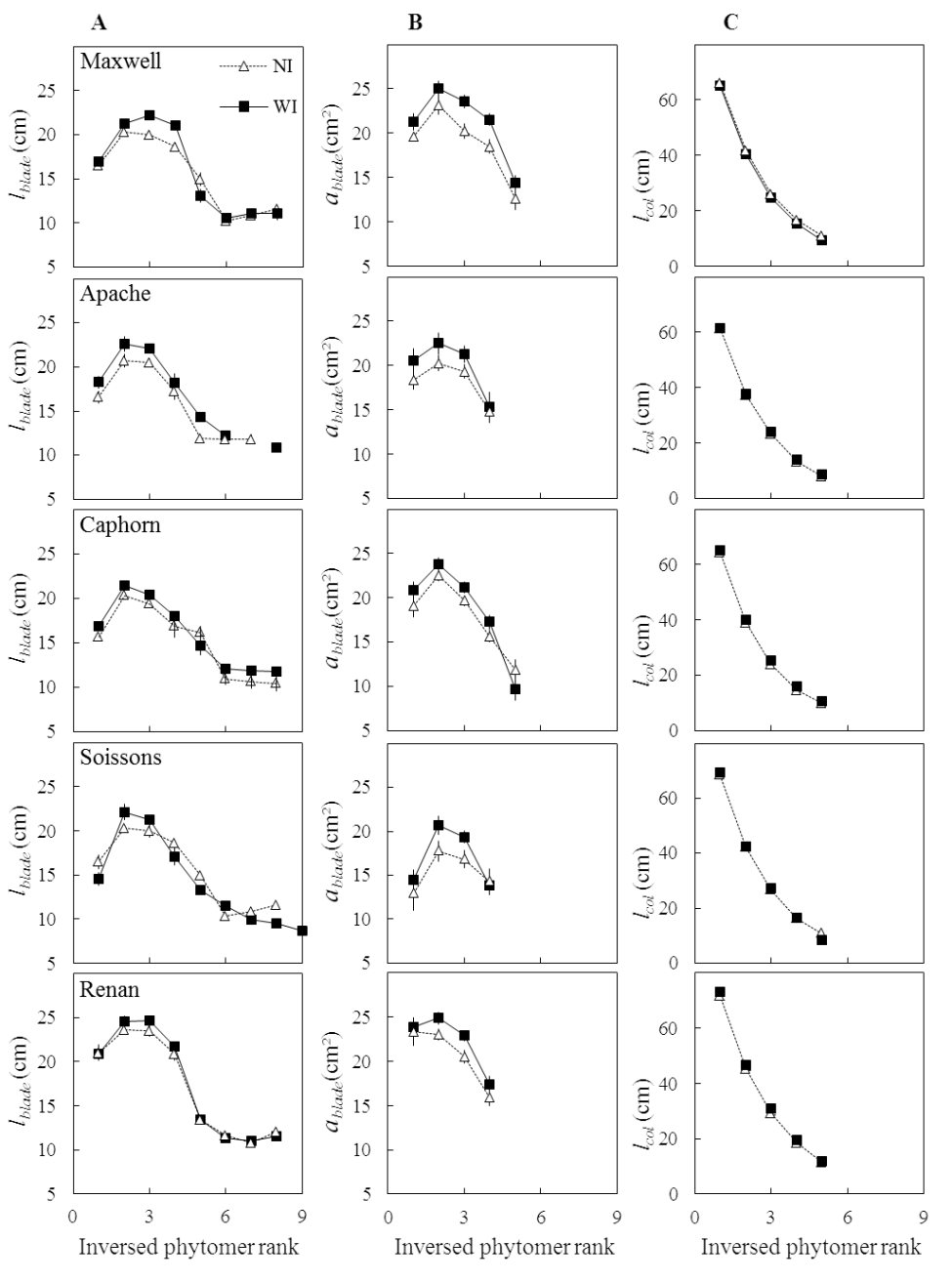


**Supplementary Figure S2** | Sequence of phytomer traits at successive positions along the main stem, for winter wheat cultivars Maxwell, Apache, Caphorn, Soissons and Renan, in NI (empty triangles) and WI (filled squares) treatments : blade length (A), blade area (B), distance from plant base to leaf collar (C). Phytomers were counted basipetally, with position 1 corresponding to the flag leaf. Vertical bars mark the 95% confidence intervals of the mean estimates.


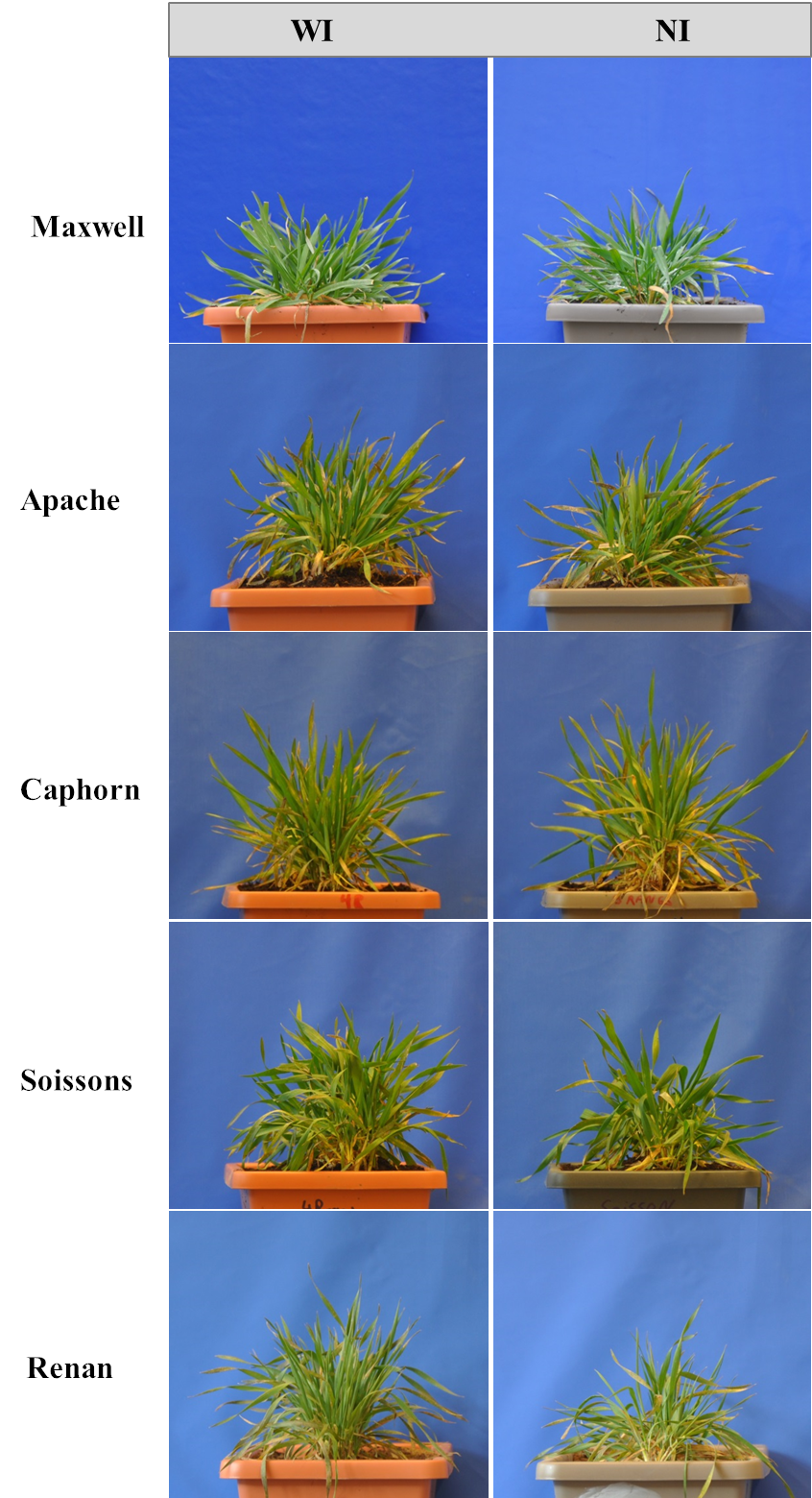


**Supplementary Figure S3** | Photographs of segments of rows collected from the field at 722°Cd after emergence and transplanted in pots to be digitized in the laboratory. Left column shows WI treatment and right column shows NI treatment, for winter wheat cultivars: Maxwell, Apache, Caphorn, Soissons, and Renan.


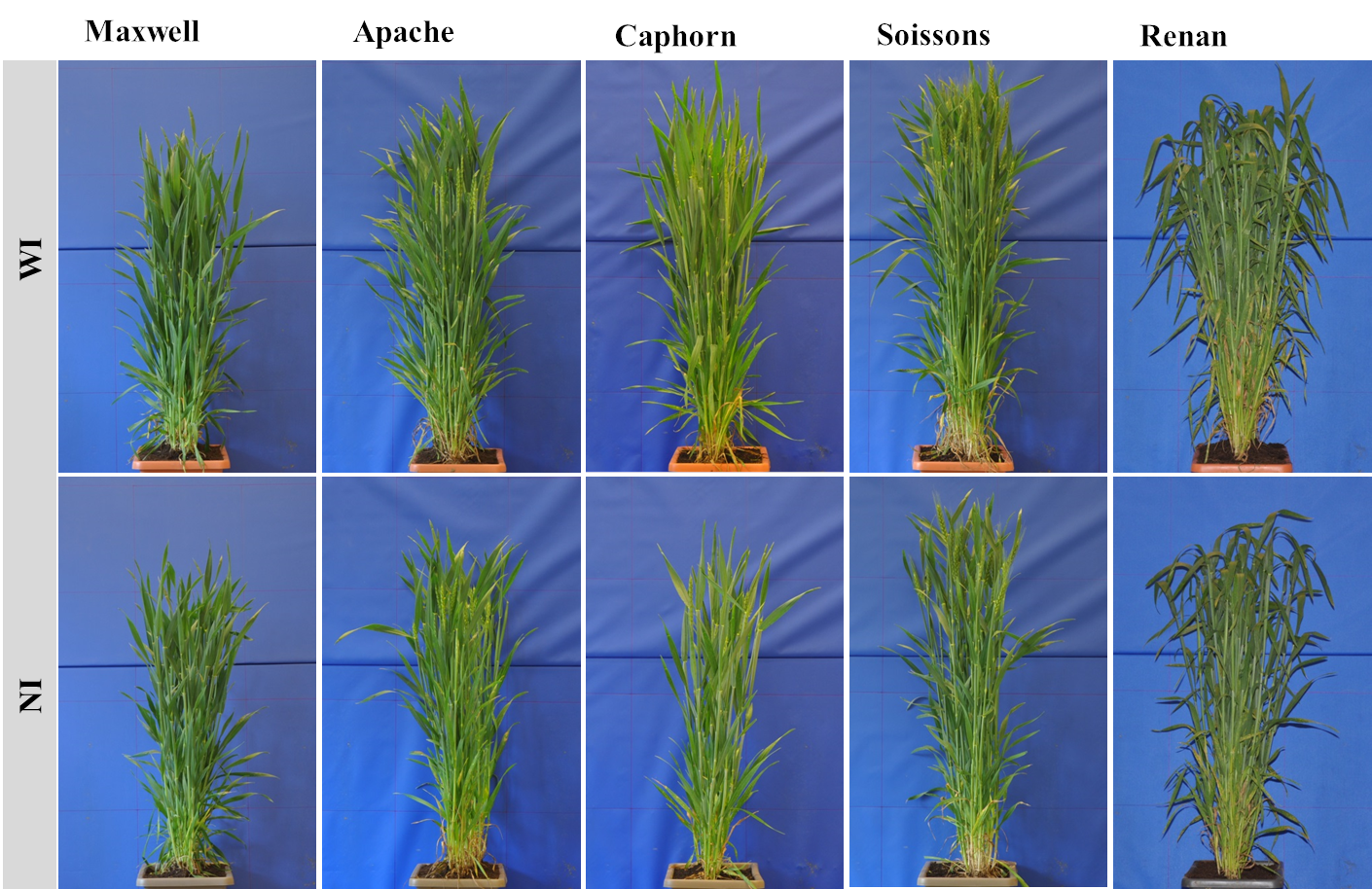


**Supplementary Figure S4** | Photographs of segments of rows collected from the field at 1272°Cd after emergence and transplanted in pots to be digitized in the laboratory. Upper line shows WI treatment and bottom line shows NI treatment, for five wheat cultivars: Maxwell, Apache, Caphorn, Soissons, and Renan.

**Supplementary Table S1** | Effect of increasing row spacing on architecture traits of winter wheat cultivars Maxwell, Apache, Caphorn, Soissons and Renan. The left part of the table shows the mean values of traits in WI and NI treatments. The right part of the table shows the outputs of tests for significance of differences between WI and NI.

| **a)** |  |  | | |  |  |  |  | | | | |  | | |  | | |  |  | | | |  | | |  | | |  | |  |
| --- | --- | --- | --- | --- | --- | --- | --- | --- | --- | --- | --- | --- | --- | --- | --- | --- | --- | --- | --- | --- | --- | --- | --- | --- | --- | --- | --- | --- | --- | --- | --- | --- |
| **Traits** | Thermal time  (°Cd) | **Mean values** | | |  |  |  |  | | | | |  | | |  | | |  | **t.test p-values** | | | |  | | |  | | |  | |  |
|  |  | Maxwell | | Apache | | Caphorn | | Soissons | | | | | | | | Renan | | | | Maxwell | Apache | | | Caphorn | | | Soissons | | | Renan | |  |
|  |  | NI | WI | NI | WI | NI | WI | | NI | | WI | | | NI | | | WI | | |  | | |  | | |  | |  | | |  | |
| Final leaf number of MS | - | 11.3 | 11.7 | 10.2 | 10.6 | 11.4 | 10.8 | | | 11.3 | | 11.7 | | | 11.0 | | | 11.4 | | 0.21 | | 0.17 | | | **0.01** | | **0.08** | | **0.04** | | |  |
| Number of elongated internodes | - | 4.37 | 4.60 | 4.30 | 4.27 | 4.48 | 4.76 | | | 4.70 | | 4.90 | | | 4.62 | | | 4.60 | | **0.073** | | 0.779 | | | **0.031** | | **0.081** | | 0.873 | | |  |
| Maximum number of axes | - | 4.7 | 3.8 | 4.7 | 3.8 | 4.3 | 3.8 | | | 5.2 | | 4.2 | | | 4.6 | | | 4.1 | | **0.05** | | **0.01** | | | 0.22 | | **0.02** | | 0.21 | | |  |
| Final area (cm²) of flag leaf | - | 19.6 | 21.3 | 18.4 | 20.6 | 19.1 | 20.9 | | | 12.9 | | 14.4 | | | 23.4 | | | 23.8 | | **0.07** | | **0.01** | | | **0.07** | | 0.19 | | 0.62 | | |  |
| 2^nd^ leaf | - | 23.2 | 25.0 | 20.3 | 22.5 | 22.5 | 23.8 | | | 17.8 | | 20.7 | | | 23.0 | | | 24.8 | | **0.01** | | **0.00** | | | **0.03** | | **0.00** | | **0.00** | | |  |
| 3^d^ leaf | - | 20.2 | 23.6 | 19.3 | 21.3 | 19.7 | 21.2 | | | 16.8 | | 19.4 | | | 20.5 | | | 22.9 | | **0.00** | | **0.00** | | | **0.01** | | **0.00** | | **0.00** | | |  |
| 4^th^ leaf | - | 18.5 | 21.5 | 14.8 | 15.3 | 15.7 | 17.3 | | | 14.3 | | 13.8 | | | 15.9 | | | 17.4 | | **0.00** | | 0.63 | | | **0.02** | | 0.62 | | **0.08** | | |  |
| *l_apex_* of the main stem | 870 | 1.6 | 1.9 | 3.6 | 4.8 | 4.1 | 5.1 | | | 5.7 | | 6.2 | | | 4.0 | | | 4.6 | | **0.02** | | **0.00** | | | **0.00** | | **0.03** | | **0.04** | | |  |
| *l_col(1)_* of the main stem | 870 | 9.9 | 10.9 | 12.1 | 14.2 | 13.0 | 14.3 | | | 15.6 | | 16.4 | | | 13.3 | | | 14.4 | | **0.00** | | **0.00** | | | **0.00** | | **0.00** | | **0.00** | | |  |
| *l_col(1)_* of the main stem | 2287 | 65.0 | 66.0 | 61.3 | 61.4 | 64.5 | 65.0 | | | 68.3 | | 69.5 | | | 71.5 | | | 73.0 | | 0.24 | | 0.89 | | | 0.62 | | 0.31 | | 0.20 | | |  |
| *i_leaf_* of the 4th upper leaves | 961 | 30.5 | 27.4 | 28.5 | 20.6 | 19.5 | 18.4 | | | 17.9 | | 18.7 | | | 20.4 | | | 23.9 | | 0.37 | | **0.007** | | | 0.61 | | 0.67 | | 0.316 | | |  |
|  | 1091 | 15.1 | 12.9 | 13.2 | 11.0 | 13.7 | 11.5 | | | 13.6 | | 13.9 | | | 9.7 | | | 8.6 | | 0.15 | | 0.22 | | | 0.21 | | 0.83 | | 0.367 | | |  |
|  | 1193 | 12.5 | 13.5 | 13.9 | 15.6 | 13.8 | 13.4 | | | 16.3 | | 15.7 | | | 12.68 | | | 10.16 | | 0.41 | | 0.30 | | | 0.73 | | 0.63 | | **0.002** | | |  |
|  | 1257 | 18.1 | 17.8 | 18.2 | 18.7 | 15.1 | 15.9 | | | 22.5 | | 21.1 | | | 17.2 | | | 13.5 | | 0.78 | | 0.69 | | | 0.51 | | 0.50 | | **0.006** | | |  |
|  | 1505 | 27.9 | 31.4 | 22.8 | 30.6 | 19.0 | 18.8 | | | 25.9 | | 30.3 | | | 18.1 | | | 18.5 | | **0.078** | | **0.001** | | | 0.90 | | **0.073** | | 0.780 | | |  |
|  | 1888 | 39.3 | 47.3 | 32.9 | 38.9 | 20.0 | 20.9 | | | 67.2 | | 63.5 | | | na | | | na | | **0.020** | | **0.066** | | | 0.52 | | 0.62 | | na | | |  |
|  | 2009 | 40.6 | 53.5 | 45.4 | 78.2 | 21.9 | 26.0 | | | 77.9 | | 104.2 | | | 27.1 | | | 34.9 | | **0.006** | | **0.000** | | | **0.069** | | **0.001** | | **0.073** | | |  |
| *d_stem_* | 722 | 1.99 | 1.50 | 1.77 | 1.68 | 1.42 | 1.65 | | | 2.27 | | 1.92 | | | 1.75 | | | 1.41 | | **0.000** | | 0.491 | | | 0.107 | | **0.036** | | **0.015** | | |  |
| *θ_stem_* | 722 | 43.4 | 44.2 | 49.9 | 47.0 | 41.2 | 46.4 | | | 47.8 | | 45.9 | | | 45.9 | | | 37.9 | | 0.831 | | 0.446 | | | 0.208 | | 0.626 | | **0.081** | | |  |
| *d_leaf_* | 722 | 7.52 | 7.28 | 7.31 | 7.20 | 6.96 | 6.76 | | | 7.64 | | 7.00 | | | 7.72 | | | 8.14 | | 0.497 | | 0.753 | | | 0.557 | | **0.088** | | 0.232 | | |  |
| *θ_leaf_* | 722 | 46.9 | 45.4 | 48.7 | 49.0 | 43.0 | 44.1 | | | 44.0 | | 46.9 | | | 48.8 | | | 42.9 | | 0.592 | | 0.904 | | | 0.680 | | 0.306 | | **0.066** | | |  |
| *d_stem_* | 1272 | 5.95 | 5.30 | 5.98 | 7.34 | 5.82 | 7.12 | | | 6.10 | | 9.01 | | | 7.83 | | | 7.85 | | 0.372 | | **0.062** | | | 0.112 | | **0.000** | | 0.982 | | |  |
| *θ_stem_* | 1272 | 41.8 | 40.5 | 36.5 | 52.8 | 49.8 | 45.2 | | | 48.7 | | 43.5 | | | 39.4 | | | 40.0 | | 0.810 | | **0.001** | | | 0.438 | | 0.341 | | 0.908 | | |  |
| *d_leaf_* | 1272 | 9.40 | 9.50 | 10.82 | 12.39 | 8.88 | 10.23 | | | 10.28 | | 13.05 | | | 11.82 | | | 12.68 | | 0.814 | | **0.010** | | | **0.007** | | **0.000** | | 0.157 | | |  |
| *θ_leaf_* | 1272 | 40.5 | 41.8 | 43.5 | 42.6 | 43.7 | 43.3 | | | 43.1 | | 41.7 | | | 44.8 | | | 42.9 | | 0.565 | | 0.734 | | | 0.903 | | 0.572 | | 0.458 | | |  |
| **b)** |  |  |  |  |  |  |  | | |  | |  | | |  | | |  | |  | |  | | |  | |  | |  | | |  |
|  |  | **Mean values** | | | | | | | | | | | | | | | | | | **Mann & Whitney (alpha s)** | | | | | | | | | | | |  |
|  |  | Maxwell |  | Apache |  | Caphorn |  | | | Soissons | |  | | | Renan | | |  | | Maxwell | | Apache | | | Caphorn | | Soissons | | Renan | | |  |
|  |  | NI | WI | NI | WI | NI | WI | | | NI | | WI | | | NI | | | WI | |  | |  | | |  | |  | |  | | |  |
| Ear number |  | 2.5 | 2.3 | 3.0 | 2.8 | 2.7 | 2.6 | | | 3.4 | | 3.0 | | | 3.1 | | | 2.8 | | 0.35 | | 0.20 | | | 0.35 | | 0.50 | | 0.35 | | |  |
| PAIg at flowering |  | 5.5 | 4.5 | 4.4 | 3.6 | 3.8 | 3.6 | | | 4.0 | | 4.0 | | | 4.6 | | | 5.0 | | 0.20 | | 0.20 | | | 0.50 | | 0.50 | | 0.35 | | |  |
